# Hidden markov modeling of emotional state transitions in interactive installation art

**DOI:** 10.1038/s41598-025-20113-7

**Published:** 2025-10-17

**Authors:** Xiaowei Chen, Jinlei Li

**Affiliations:** 1 College of Arts, Zhejiang Shuren University, Hangzhou, China; 2 College of Creative Arts, Universiti Teknologi MARA, Shah Alam, Malaysia; 3 Zhejiang Institute of Communications, Hangzhou, Zhejiang China

**Keywords:** Hidden markov models, Interactive installation art, Emotional Well-Being, Dynamic emotional modeling, Human-Computer interaction, Human behaviour, Statistics

## Abstract

Interactive installation art provides a distinctive context for examining collective emotion, yet most prior studies have relied on laboratory or longitudinal data that are impractical in public cultural settings. This study applied Hidden Markov Models (HMMs) to self-reported well-being data from *HappyHere*, a participatory light installation at the National Galleries of Scotland. Despite the cross-sectional design, the HMM framework enabled inference of latent affective states and probabilistic differentiation patterns, stability, and convergence patterns. The results show four key findings. First, three qualitatively distinct latent states were identified: a low/negative cluster (M ≈ 1.5), a moderately positive cluster (M ≈ 3.5), and a ceiling-level, highly positive cluster (all M = 5.0), confirming clear differentiation (H1). Second, positive states proved the most stable, with the highest self-transition probability (0.875) and the longest dwell time (≈ 3.4 steps), supporting H2. Third, neutral states were comparatively unstable, showing the lowest self-transition probability (0.093) and a tendency to shift toward positivity, consistent with H3. Finally, the stationary distribution strongly favored positivity, with positive states Reaching 86.3%, neutral states 7.5%, and negative states 6.2%. Minor diurnal variation was observed, but effect sizes were negligible, confirming the enduring predominance of positivity (H4). The findings demonstrate that even cross-sectional cultural datasets can yield meaningful insights through probabilistic modelling. Positive affect emerged as the most stable and dominant attractor, underscoring the capacity of participatory art to regulate emotion and foster collective well-being.

## Introduction

Interactive installation art has become an increasingly influential medium for evoking emotional responses and fostering affective engagement. By enveloping participants in multisensory, immersive environments, such installations provide a unique context where human affect can be experienced, expressed, and examined. These participatory settings offer fertile ground for promoting emotional well-being. However, emotional experiences within them remain inherently dynamic—subject to continuous fluctuation in response to both internal psychological processes and external environmental stimuli. Understanding these variations requires modelling methods to capture underlying affective patterns in ecologically valid, non-laboratory settings.

Despite growing scholarly and artistic interest in interactive media as a vehicle for emotional exploration, most studies focus on short-term emotional reactions captured through multimodal physiological or behavioural indicators such as facial expression recognition, galvanic skin response, or electroencephalography (EEG). For instance, Hidden Markov Models (HMMs) have been employed in controlled experimental settings to analyze state transitions in affective time series^1,2^, and interactive platforms have also been shown to shape user emotions^3^. However, such approaches require resource-intensive data collection and are often impractical in public art settings, where lightweight and scalable measures are needed. Our study adapts HMMs to cross-sectional data, inferring population-level latent states rather than modelling within-person temporal dynamics.

 This study addresses these challenges by using a publicly available dataset of self-reported well-being (HappyHere) to examine latent affective states in a participatory art installation. Unlike longitudinal research that follows individual trajectories, our approach applies HMMs to single-occasion, cross-sectional responses to infer population-level emotional configurations and their relative stability. Stability is operationalized via state self-transition probabilities and entropy, enabling an examination of how affective states are represented, how they vary in aggregate across participants, and which states demonstrate greater cohesion or dominance under the estimated stationary distribution. Building on this rationale, four research questions (RQs) are posed rather than formal hypotheses at the introduction stage: (RQ1) Which latent states correspond to distinct valence profiles? (RQ2) Do positive states exhibit greater stability and cohesion? (RQ3) Are neutral states more variable and dispersed? (RQ4) Which states dominate the stationary distribution? The formal hypotheses operationalizing these questions are developed in Sect. 3.

The contributions of this study are threefold. First, an adaptation of HMMs for cross-sectional self-report data is introduced as an exploratory framework for inferring latent affective states in a real-world art context. Second, the cohesion and stability of these states are evaluated and quantified by self-transition probabilities and entropy, underscoring the prominence of positive affect. Third, the methodological and design implications of applying probabilistic models to cross-sectional emotional data are outlined, offering insights for developing affect-sensitive interactive systems. These contributions align the method, hypotheses, and design takeaways within a coherent analytical logic.

## Literature review

Studying dynamic emotional states has garnered increasing interdisciplinary attention, particularly in sequential data modelling. Among the computational tools employed, Hidden Markov Models (HMMs) have demonstrated utility in capturing latent affective configurations and their probabilistic patterns^4^. For example, time-varying autoregressive (TVAR) models have been applied to detect gradual and abrupt changes in affective trajectories, thereby underscoring emotional processes’ adaptive and non-stationary nature^1^. While such methods effectively capture short-term variability, their applicability to population-level stability patterns in ecologically valid settings has not been extensively validated.

Happiness, conceptualized as a dynamic psychological construct, is modulated by emotional intensity and temporal frequency fluctuations. Meta-analytic evidence highlights the roles of emotional variability and resilience in supporting sustained subjective well-being^5^. Similarly, psychological resources—such as hope and perceived meaning in life—have been identified as stabilizing factors for positive affective states^6^. These findings reinforce the significance of temporal characteristics in well-being research; however, most such investigations lack computational rigour in modelling the structural distribution of emotional states beyond short-term data.

Integrating HMMs with self-reported emotional data offers a promising methodological bridge. For instance, Hidden Semi-Markov Models (HSMMs) have been employed to capture variable-duration emotional expressions in speech synthesis, enhancing the granularity of latent state interpretation^7^. However, despite these advances in methodological expressiveness, the application of such models to real-world, immersive environments remains limited—especially in the domain of interactive installation art. In particular, little attention has been paid to how self-reported affective states cluster and stabilize at the group level in participatory, aesthetic experiences.

Three primary limitations characterize current research. First, the dominant focus is on short-term responses, with limited examination of population-level state distributions^8–10^. Second, while HMMs have been widely adopted in clinical and experimental psychology, their integration into experiential and user-centred contexts—such as participatory art installations—is scarce^2^. Third, although self-reported affective data is cost-effective, scalable, and well-suited to public-facing environments, it is underutilized in dynamic modelling frameworks capable of capturing sequential emotional patterns^11^. These gaps highlight the need for an experience-centred, exploratory approach to modelling emotional dynamics in participatory settings.

The present study addresses these gaps by applying HMMs to a publicly available dataset from an interactive art installation. This approach identifies latent affective states and examines their relative stability. It provides methodological and design insights while recognizing that cross-sectional HMM applications capture group-level probabilistic patterns rather than individual longitudinal trajectories.

## Theoretical framework

This study is grounded in an interdisciplinary theoretical framework integrating computational modelling techniques, psychological theories of affective dynamics, and interaction design principles. The framework aims to explicate the mechanisms underlying latent emotional configurations and their relative stability within interactive installation art. Accordingly, the framework encompasses three interconnected components: (1) population-level emotional modelling with Hidden Markov Models (HMMs), (2) psychological mechanisms of affective stability and variability, and (3) the regulatory influence of interactive system design.

The first component emphasizes using HMMs as a probabilistic tool for modelling latent emotional states and their transitions over time^12^. In this study, rather than reconstructing within-person longitudinal trajectories, HMMs reveal population-level regularities in the distribution of affective states. This exploratory approach enables the identification of probabilistic patterns in emotional dynamics, even when data are cross-sectional rather than longitudinal.

The second component is grounded in the broaden-and-build theory^13^, which posits that positive emotions expand cognitive and social resources, enhancing psychological stability^14^. At the collective level, positive affect fosters cognitive convergence^15^ and supports the emergence of interference-resistant social networks^16^. These benefits may be moderated by contextual factors such as group identity and emotional intensity^17,18^. Contrastingly, neutral or ambivalent states are often more volatile, displaying a greater propensity to fluctuate across contexts. This body of evidence provides the theoretical foundation for interpreting the relative persistence of latent affective states according to their valence.

The third component highlights the role of interaction design as a mechanism for emotional regulation. Through real-time feedback (e.g., motion capture) and multisensory stimulation (e.g., audiovisual–tactile integration), interactive installations can function as immersive platforms that reinforce positive affective patterns, including empathy and prosocial engagement^19^. Furthermore, by incorporating biofeedback techniques that directly modulate physiological signals, such systems can significantly attenuate negative affective trajectories^20^. These considerations suggest that features of interactive environments can shape collective emotional distributions, fostering the emergence of cohesive positive states.

Building on this framework and the exploratory application of HMMs to cross-sectional data, four hypotheses were formulated, each positioned at the population level to reflect that the HappyHere dataset consists of single, anonymous submissions rather than longitudinal trajectories.

### Hypothesis 1

(H1): Emotional State Differentiation. Emotional valence will be associated with distinct latent states in the HMM. Neutral states are expected to exhibit higher entropy, reflecting greater variability in state occupancy, whereas positive states should demonstrate stronger internal consistency (e.g., higher reliability indices or lower entropy values). This expectation is consistent with methodological foundations of HMM-based state modelling^12^ and supported by empirical evidence showing that positive affective constructs yield stronger internal consistency, while neutral states are characterized by greater dispersion^21^.

### Hypothesis 2

(H2): Positive State Stability. Positive states will demonstrate higher self-transition probabilities, with robustness confirmed through bootstrap confidence intervals. This prediction aligns with the broaden-and-build theory, which suggests that positive emotions foster resilience by accumulating psychological resources^22^. Methodologically, it is consistent with evidence that latent constructs linked to positive affect maintain stability across groups in bootstrap-based models^23^.

### Hypothesis 3

(H3): Instability of Neutral States. Neutral or mixed states will correspond to higher entropy and lower self-transition probabilities, indicating instability and vulnerability to dispersion. Prior modelling studies have reported small negative paths that highlight destabilization under conflicting influences, and psychological research likewise shows that neutral or ambivalent states are particularly prone to variability^24^. Evidence from environmental psychology further suggests that competing situational demands more easily disrupt such states^25^.

### Hypothesis 4

(H4): Prevalence of Positive States. In the stationary distribution, positive states are expected to dominate. This expectation is consistent with meta-analytic findings linking short-term affective dynamics to overall well-being^10^ and parallels research in organizational and innovation contexts, where systemic favorable conditions explain most behavioral variance^26^. Additionally, reviews of subjective well-being underscore the resilience and prevalence of positive affective patterns across populations^27^.

In sum, this theoretical framework provides a comprehensive and integrative basis for investigating latent emotional patterns in interactive art environments. It facilitates the empirical interpretation of affective states and establishes a computationally grounded foundation for designing emotionally adaptive systems. By bridging psychological theory, probabilistic modelling, and interaction design, the framework advances understanding of how immersive environments can be structured to promote sustained emotional well-being.

## Research design

This study employed Hidden Markov Models (HMMs) to examine population-level latent emotional configurations derived from self-reported data collected through the HappyHere interactive art installation. HappyHere, a participatory and multisensory environment, was designed to foster individual emotional introspection and collective reflection on well-being^28^. The installation captured participants’ emotional states by integrating tactile input, auditory cues, and generative visual feedback while providing immediate, adaptive responses.

Each participant provided an anonymous submission based on the Short Warwick-Edinburgh Mental Well-Being Scale (SWEMWBS), Reflecting self-reported emotional experiences over the preceding two weeks. No longitudinal tracking was implemented, and all Records were cleared after submission; therefore, the dataset is cross-sectional rather than longitudinal. Accordingly, HMMs were applied in an exploratory, population-level manner to infer latent affective states and to assess their relative stability and prevalence, rather than to model within-person temporal change. Data collection occurred between December 2018 and February 2019 at the National Galleries of Scotland.

**Fig. 1:**
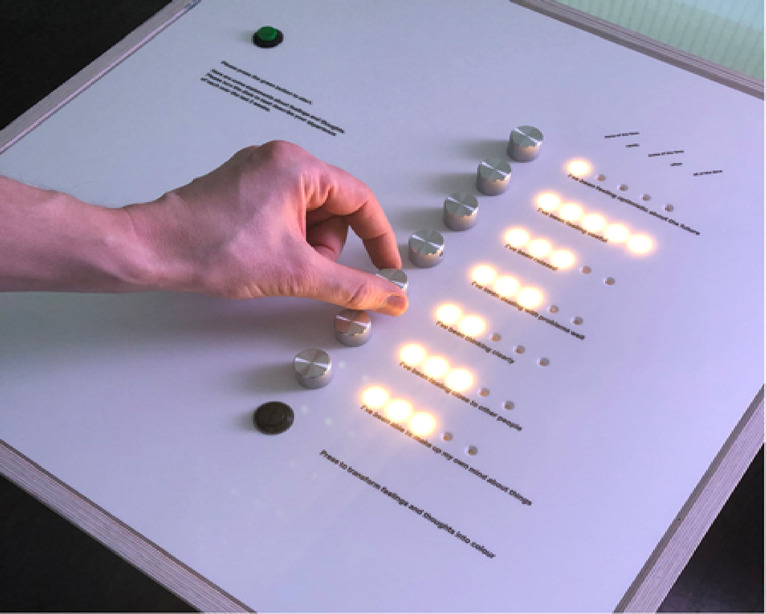
Input console used for emotional self-reporting. *Reproduced with permission from Skelly & Thomas (2024)*^28^.

**Fig. 2:**
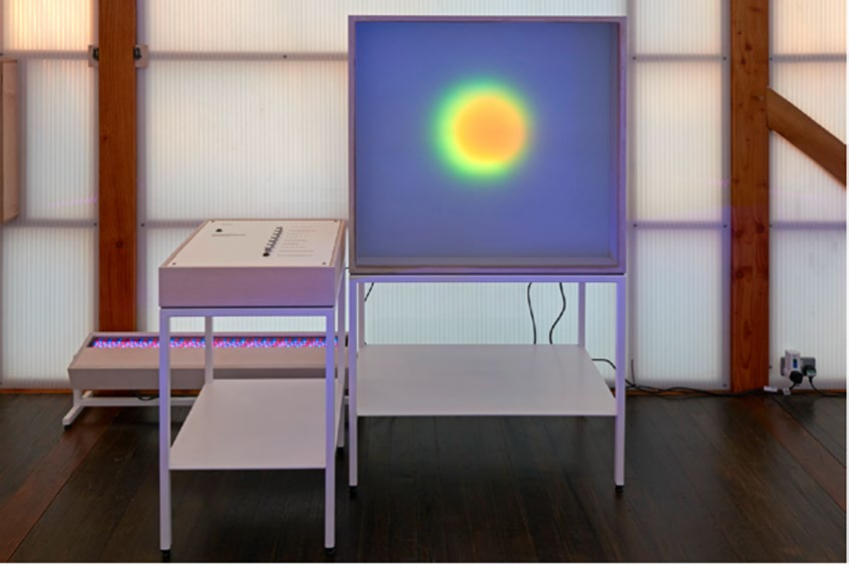
Output display showing swirling visuals mapped to emotional data. *Reproduced with permission from Skelly & Thomas (2024)*^28^.

**Fig. 3:**
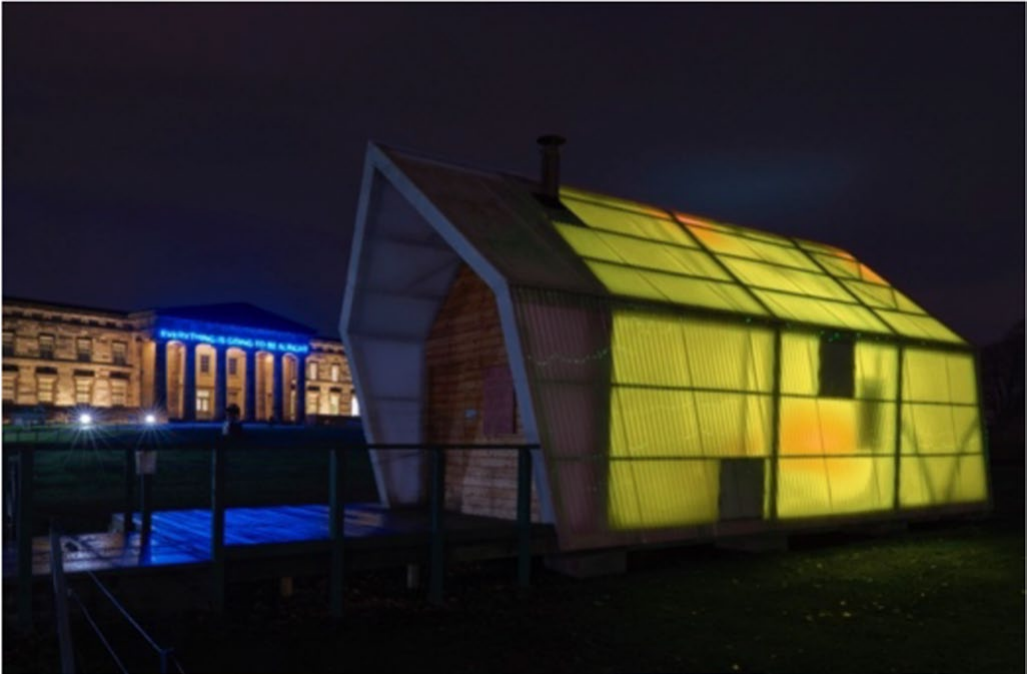
Aggregated emotional state visualization during night mode. *Reproduced with permission from Skelly & Thomas (2024)*^28^.

This design provided a rich and ecologically valid dataset as a naturalistic testbed for investigating probabilistic affective structure, thereby motivating the modelling framework elaborated in the following sections. The installation comprised three principal components that supported both data acquisition and the visualization of emotional states (Figs. 1, 2 and 3):

- Input Console: Participants interacted anonymously with a tactile interface based on the Short Warwick-Edinburgh Mental Well-Being Scale (SWEMWBS), a validated measure of emotional well-being. Responses to seven items were Rated on a 5-point Likert scale ranging from “None of the time” to “All of the time,” reflecting emotional experiences over the past two weeks. To preserve participant anonymity, all entries were automatically cleared after submission. This structured input method ensured consistency across users and enhanced the validity of emotional data used for sequential modelling.

- Output Display: Upon submission, the installation rendered the emotional profile as generative, swirling light patterns that gradually converge into a static colour field. This process provided immediate visual feedback, offering an introspective moment that links subjective emotional states to dynamic visual expression. By visually encoding affective states, this component reinforced the self-reporting’s emotional salience and contextualized emotional data within a sensory feedback loop.

- Night Mode Visualization: In the evening, the system aggregates daily data into a unified ambient light projection visible outside the gallery. This collective display transformed individual responses into a shared emotional atmosphere, underscoring the installation’s role as a socially engaged, public-facing intervention. This nightly aggregation offered macroscopic insights into community-level affective rhythms, extending the model’s interpretability to collective emotional dynamics. Importantly, no new self-report submissions were collected during evening hours, as the installation’s “night mode” replayed data gathered earlier in the day rather than recording new inputs. Therefore, all statistical analyses in this study are based exclusively on daytime submissions. (Galleries were open to the public daily from 10:00 to 17:00 during the exhibition period.)

### Data preparation and modeling approach

Before modelling, we used the publicly available HappyHere dataset^29^, collected at the National Galleries of Scotland (Mendeley Data, V1, 10.17632/tpcwm8d7d3.1). The dataset underwent standard preprocessing procedures, including timestamp normalization, rescaling of SWEMWBS scores, and exclusion of incomplete entries. These steps ensured data integrity and suitability for sequential probabilistic modelling. HMMs were selected for their ability to infer latent (unobservable) emotional states and to estimate transition probabilities over time. Within this framework, SWEMWBS item scores constituted the observable emissions, while the underlying affective states were modelled as hidden variables. The HMM consists of the following probabilistic components that govern state behaviour and observations:

- Transition Matrix: This matrix encodes the probabilities of transitioning between latent emotional states, capturing temporal stability or volatility in affective dynamics.
- Emission Probabilities: Define the likelihood of observed responses given a particular latent state, linking reported emotions to probabilistic effect representations.
- Initial State Distribution: Specifies the likelihood of entering each latent state, enabling inference of baseline affective conditions at the outset of interaction.

The number of hidden states was determined using the Bayesian Information Criterion (BIC), which balances model fit and complexity. This approach minimizes the risk of overfitting, which is significant in studies with finite sample sizes and noisy self-reported data. Model training was conducted using the Baum-Welch algorithm, a variant of the Expectation-Maximization (EM) method, to estimate optimal transition and emission parameters iteratively. The dataset was collected initially under ethical approval from the University of Dundee, DJCAD, School of Art and Design Research Ethics Committee (Approval ref: DJCAD_SDAD_19_AS0137). Less than 0.1% of Responses were Recorded as 0 or − 1, outside the intended 1–5 Likert scale. These were treated as missing values and excluded from descriptive analyses.

### Visualization and interpretation

A suite of visualizations was generated to support interpretation of the HMM outputs, including: Heatmaps of transition probabilities to identify recurrent and stable state paths, Temporal plots of state distributions to observe evolving emotional profiles, and Convergence trajectories to examine long-term emotional stability and dominant states. These visual tools facilitated a comprehensive analysis of effective fluctuation, state persistence, and emotional trajectory convergence. Through this modeling approach, the study aimed to generate empirically grounded insights into affect-aware design practices in interactive systems. These visual analysed serve not only to validate the modelling framework but also to inform the design of adaptive emotional interventions in interactive art settings.

### Statistical analyses

To complement the HMM modelling framework, a series of statistical analyses was conducted to characterize the dataset and evaluate population-level patterns:

- Descriptive statistics were calculated for each SWEMWBS item (means, standard deviations, minimum, maximum, and quartiles). These values provide context for the affective landscape of the dataset and allow comparison with the latent states inferred by the HMM.
- Chi-square tests of independence were performed to examine whether the distribution of hidden states differed across time bins (morning: 06:00–12:00; afternoon: 12:00–18:00). Test results were reported with χ² statistics, degrees of freedom, and p-values. Effect sizes were quantified using Cramer’s V, with thresholds of 0.1, 0.3, and 0.5 interpreted as slight, moderate, and significant effects, respectively.
- State entropy was computed to quantify the variability of each latent state, with higher entropy indicating greater dispersion in state occupancy. Entropy measures provide a complementary index of stability alongside transition probabilities.
- Dwell time was calculated as the expected number of steps in which participants’ data remained in a given state before transitioning to another. Shorter dwell times indicate transient states, while longer dwell times suggest persistence.
- Bootstrap resampling (1,000 iterations) was used to estimate confidence intervals for key transition probabilities, ensuring robustness of the stability and convergence estimates reported in the Results. Because SWEMWBS responses are ordinal Likert-type scores and unlikely to follow a normal distribution, bootstrap resampling was chosen as a non-parametric approach that does not require normality assumptions.

Together, these analyses enabled descriptive and inferential evaluation of the latent states identified by the HMM, grounding the exploratory modelling in statistical evidence and effect-size interpretation.

## Results

This section presents the outcomes of the Hidden Markov Model (HMM) analysis conducted on the HappyHere dataset, focusing on the distribution, transition dynamics, and long-term convergence of latent emotional states.

### Descriptive statistics and emotional context

Table 1 presented the descriptive statistics for key self-reported emotional indicators after excluding a minimal number of anomalous responses (0 or − 1). The distributions aligned with the expected 1–5 Likert scale. Positive indicators such as optimism (M = 3.50), social connectedness (“close people,” M = 3.53), and self-consistency (“own mind,” M = 3.53) showed relatively high mean values, indicating a general tendency toward positive affect across participants. Similarly, usefulness (M = 3.22) and clarity (M = 3.19) also exhibited moderately high averages. These descriptive statistics provided the empirical backdrop for interpreting the affective landscape of the dataset and were consistent with the high prevalence of State 2 observed in the HMM analysis. The correspondence between these positively valenced indicators and the stable, frequent occurrence of State 2 further supported its interpretation as a predominantly positive configuration.

**Table 1:** Descriptive statistics of the emotional variables Observed.

Variable	Mean	Std	Min	25%	50%	75%	Max
Optimistic	3.50	1.25	1	3.0	4.0	4.0	5
Useful	3.22	1.31	1	2.0	3.0	4.0	5
Relaxed	3.03	1.35	1	2.0	3.0	4.0	5
Problems	3.17	1.31	1	2.0	3.0	4.0	5
Clearly	3.19	1.31	1	2.0	3.0	4.0	5
Close People	3.53	1.39	1	3.0	4.0	5.0	5
Own Mind	3.53	1.37	1	3.0	4.0	5.0	5

### Emotional state differentiation

Table 2 presented descriptive statistics for the observed well-being indicators across the three HMM-inferred latent states. State 0 (moderately positive) was associated with mid-to-high scores on optimism, usefulness, and social connection, suggesting a favorable but not extreme affective profile. In contrast, State 1 (low/negative) was marked by uniformly low scores across all indicators, Reflecting a consistently negative configuration. Finally, State 2 (highly positive) corresponded to ceiling-level responses, indicating strongly positive affective experiences.

**Table 2:** Descriptive means of observed variables by State.

State ID	Optimistic	Useful	Relaxed	Problems	Clearly	Close People	Own Mind
0 (Moderately Positive)	3.55	3.24	3.02	3.19	3.21	3.60	3.60
1 (Low/Negative)	1.63	1.48	1.48	1.48	1.48	1.48	1.48
2 (Highly Positive)	5.00	5.00	5.00	5.00	5.00	5.00	5.00

*Note: Table reports mean values of self-reported well-being indicators (SWEMWBS items) within each latent state. State 2 corresponds to ceiling-level positive responses*,* State 1 to consistently low affect*,* and State 0 to moderate positive affect.*

**Fig. 4:**
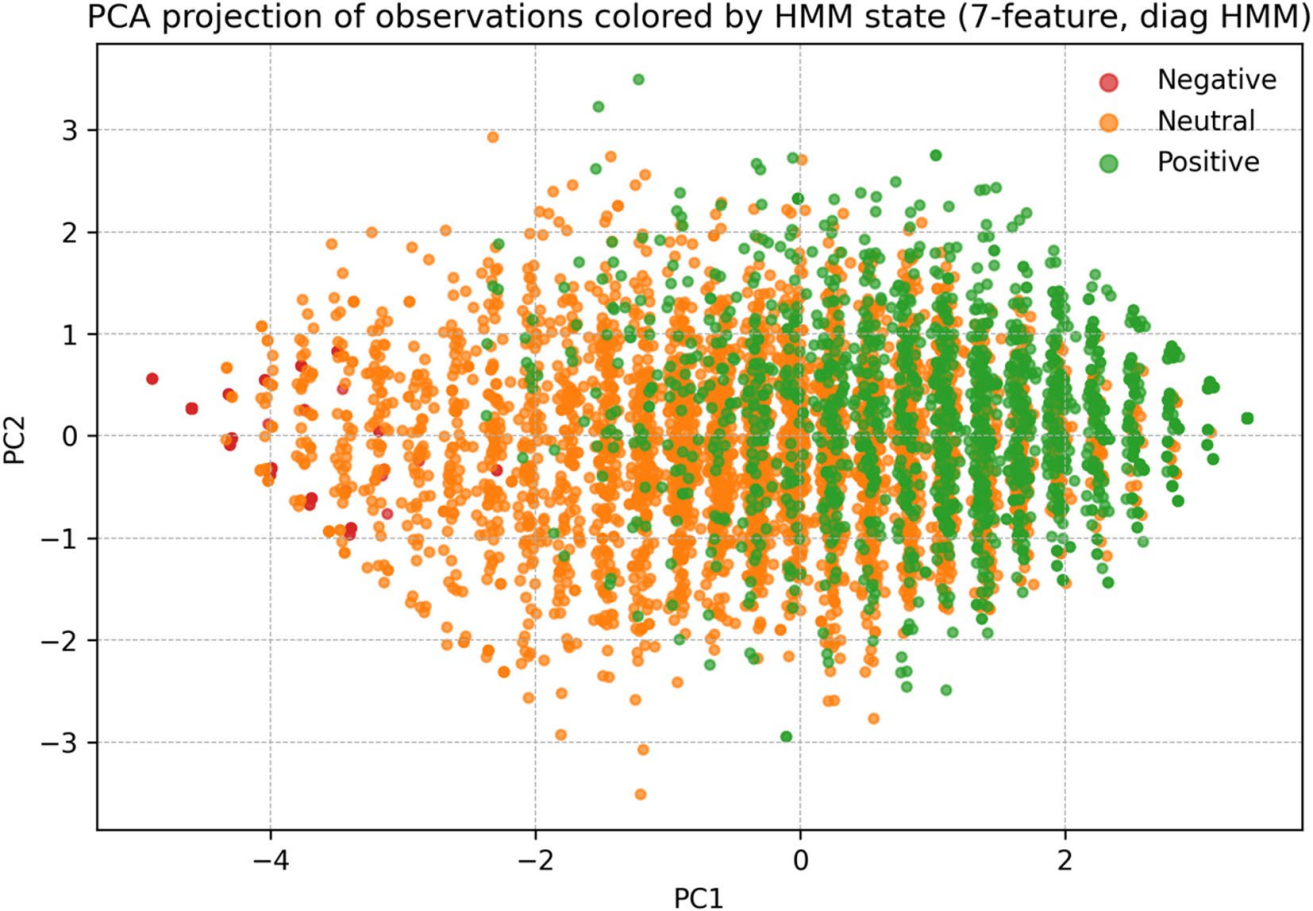
PCA projection of observations colored by HMM state.

These patterns demonstrated clear differentiation among the latent states, thus providing strong support for H1. Dimensionality-reduction analyseed (PCA/t-SNE; Fig. 4) further confirmed that the states formed coherent and separable clusters in reduced feature space.

To further validate these distinctions, mean scores of the seven indicators were directly compared across states. The results revealed three non-overlapping affective profiles: State 1 represented a consistently negative profile (M ≈ 1.48–1.63), State 0 reflected a moderate or mixed profile (M ≈ 3.0–3.6), and State 2 corresponded to an extreme positive cluster with ceiling-level responses (all M = 5.0). These contrasts indicated that the HMM-derived states are psychologically interpretable rather than arbitrary statistical groupings.

### Transition dynamics and stability

**Fig. 5:**
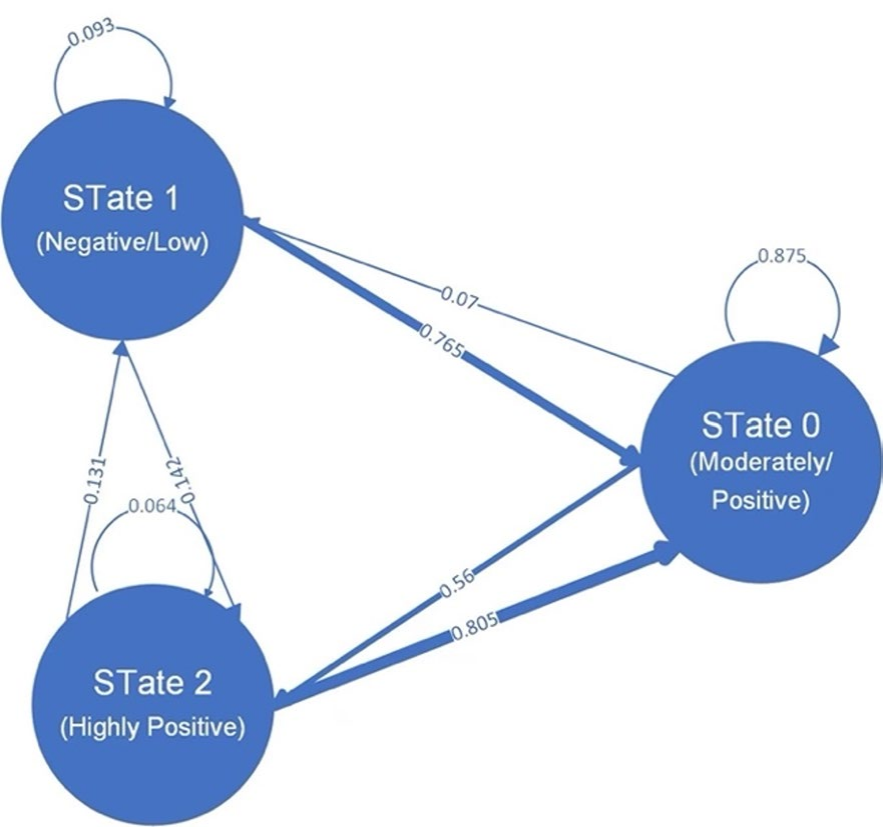
Transition Matrix of Latent Emotional States (7-feature, full-covariance HMM).

The transition dynamics of the HMM are summarized in Table 3; Fig. 5. State 0 (moderately positive/neutral) exhibited the highest self-transition probability (0.875), Reflecting a Relatively stable configuration. In contrast, State 1 (low/negative) showed the lowest self-transition probability (0.093), underscoring its volatility. State 2 (highly positive) also demonstrated strong persistence, with a self-transition probability of 0.805. Nonetheless, it retained a modest probability of transitioning into the negative state (0.064), suggesting that although generally stable, it remained somewhat susceptible to contextual disruption. Bootstrap resampling confirmed the robustness of these probability estimates. Participants remained longest in positive states (expected dwell time ≈ 3.4 steps), compared with ≈ 1.8 steps in neutral states and ≈ 1.3 steps in negative states, further reinforcing the persistence of positive affective configurations.

**Table 3:** Transition matrix of latent emotional States (7-feature HMM).

From/To	State 0 (Moderately Positive/neutral)	State 1 (Low/Negative)	State 2 (Highly Positive)
State 0	0.875	0.067	0.058
State 1	0.093	0.451	0.456
State 2	0.064	0.131	0.805

*Note: Transition probabilities are Rounded to three decimals; Row sums add up to approximately 1 due to rounding.*

**Fig. 6:**
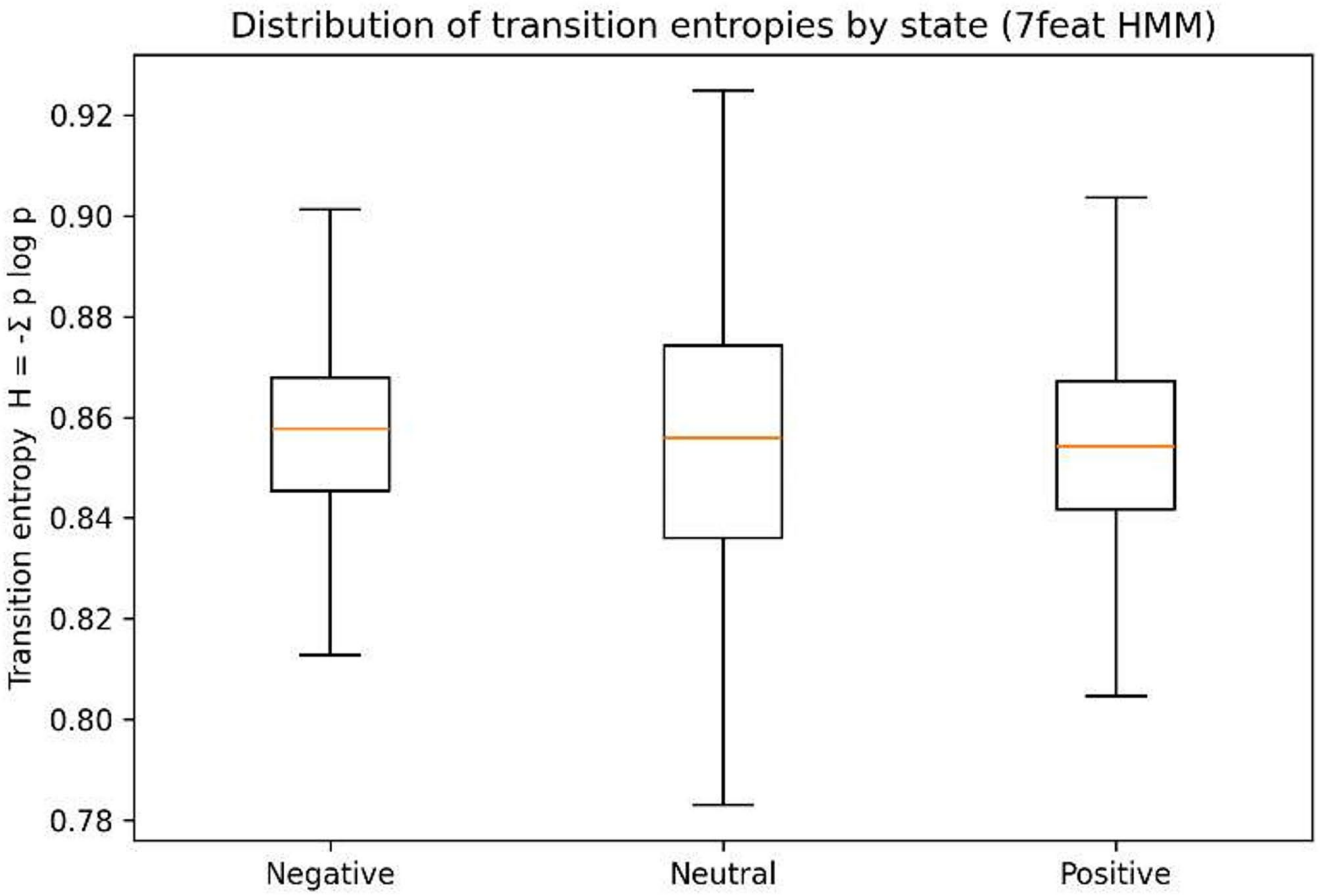
Distribution of transition entropies by state.

Shannon transition entropy (H = −Σp log p) was also computed to capture uncertainty in state transitions. Table 4; Fig. 6 show that neutral states exhibited the highest mean entropy and the widest 95% bootstrap confidence interval, indicating instability and transitional behavior. Negative states also displayed elevated entropy values but with a more minor variance, suggesting volatility without long-term persistence. By contrast, positive states combined relatively lower entropy with narrower dispersion, in line with their higher self-transition probability and longer dwell times. Overall, these findings indicated that positive states function as the most stable attractors in the system. In contrast, neutral states acted as transitional configurations prone to dispersion, and negative states were volatile but short-lived. Overall, these results strongly support H2 (stability of positive states) and H3 (instability of neutral states).

**Table 4:** Transition entropy summary (7-feature HMM).

label	mean	CI 2.5%	CI 97.5%
Negative	0.843	0.687	0.895
Neutral	0.843	0.681	0.903
Positive	0.842	0.670	0.909

### Stationary distribution of latent States

**Fig. 7:**
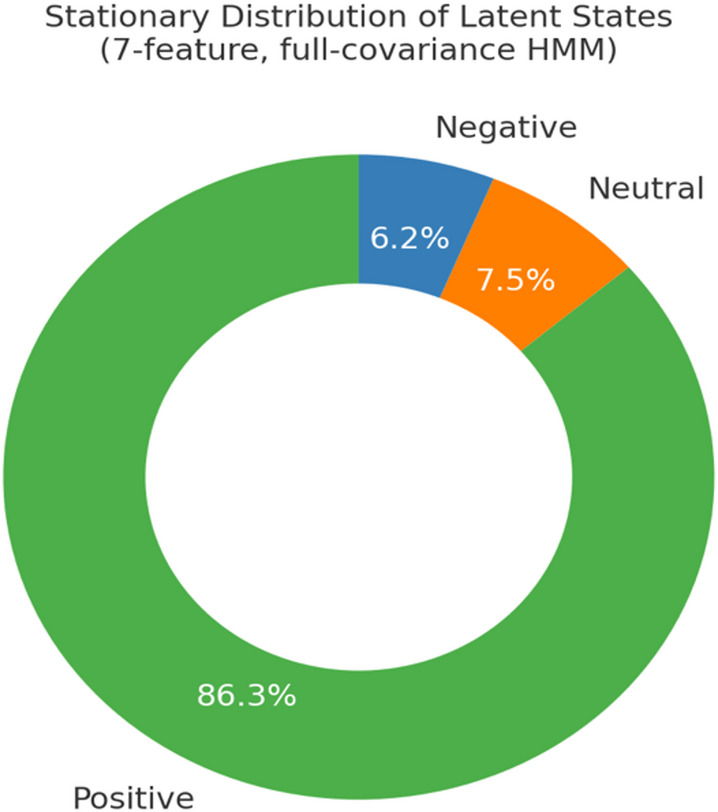
Stationary Distribution Plot.

Figure 7 presented the long-term stationary distribution of the HMM (full-covariance model, 7 features). Positive states emerged as the dominant configuration, with a steady-state probability of 86.3%. Neutral states accounted for 7.5%, and negative states for 6.2%. These proportions indicated that, in the long run, positive affect is strongly favored, and this pattern remained robust across model variants.

### Temporal variation of emotional states

To evaluate whether the distribution of emotional states varied by time of day, state distributions between morning (*N* = 1,042) and afternoon (*N* = 5,517) sessions were compared. Across both periods, positive states Remained predominant, comprising 78.8% of observations in the morning and 74.6% in the afternoon. Neutral states appeared slightly more frequently in the afternoon (16.7%) than in the morning (13.8%), whereas negative states were consistently rare across both time windows. A chi-square test of independence confirmed that these differences were statistically significant, χ²(2, *N* = 6,560) = 8.20, *p* = 0.016. Nevertheless, the effect size was negligible (Cramer’s V = 0.04), indicating that the detected fluctuations were of minimal substantive significance, although statistically reliable. Thus, diurnal differences did not materially alter our main conclusions, and the predominance of positive affect remains stable across time bins.

## Discussion

### Key findings and interpretation

This study applied Hidden Markov Models (HMMs) in an exploratory, population-level manner to infer latent affective configurations in the *HappyHere* interactive installation. Although the dataset was cross-sectional, the HMM framework added value by revealing probabilistic differentiation patterns, stability, and long-term convergence among emotional states. Four key findings emerged, corresponding to the hypotheses proposed.

First (H1: Differentiation), the HMM identified three qualitatively distinct latent states. Descriptive comparisons indicated that State 1 consistently showed the lowest scores across all indicators (e.g., optimism M = 1.63), State 0 reflected intermediate values (optimism M ≈ 3.55), and State 2 was marked by uniformly maximal scores (all items = 5.0). Analyses of entropy further indicated that neutral/mixed states were less cohesive than positive states, supporting their psychological interpretability rather than implying arbitrary statistical clustering.

Second (H2: Stability of Positive States), the transition matrix revealed that positive states possessed the highest self-transition probabilities (≈ 0.80–0.88), with bootstrap confidence intervals confirming the Robustness of these estimates. Dwell-time analyses further showed that participants Remained in positive states for an average of 3.4 steps, versus 1.8 steps for neutral and 1.3 for negative states, underscoring their greater persistence.

Third (H3: Instability of Neutral States), neutral states exhibited the lowest self-transition probability (≈ 0.09) and showed a directional tendency to transition toward positivity. Their higher entropy values supported the interpretation that neutrality functions as a transitional rather than a stable configuration and is particularly susceptible to dispersion under contextual influences.

Finally (H4: Prevalence of Positive States), the analysis was Restricted to morning and afternoon submissions because the installation entered Replay-only mode after closure, and no valid evening data were collected. The stationary distribution converged strongly toward positivity, with positive states accounting for 86.3% in the 7-feature, full-covariance model, and this pattern was robust across alternative model specifications. A chi-square test comparing morning and afternoon distributions was statistically significant, χ²(2, *N* = 6,560) = 8.20, *p* = 0.016. However, the effect size was negligible (Cramer’s V = 0.04), indicating that the predominance of positive affect remained stable across time bins despite minor diurnal variation.

### Comparison with prior research

Beyond confirming these hypotheses, our findings reveal important extensions to prior research on emotion dynamics across psychology, social media, and cultural contexts.

Differentiation of latent states. Identifying three qualitatively distinct states is consistent with a substantial body of theory and meta-analytic work indicating that emotions are not arbitrary statistical clusters but rather psychologically interpretable patterns. Houben et al.^10^ further demonstrated that variability and instability in negative affect are consistently associated with diminished well-being. Fredrickson^22^ argued that positive emotions broaden cognition and consolidate distinct experiential profiles. Similarly, Kang^4^ showed that joy, sadness, and fear could be reliably detected using HMMs in video content, underscoring the feasibility of linking low-level indicators to meaningful affective states. By recovering a three-state structure in a participatory art setting—moderately positive, low/negative, and highly optimistic—our results extend these distinctions beyond laboratory or media-based contexts and demonstrate their salience in public cultural environments.

Stability and instability of states. The observation that positive states displayed the highest self-transition probability and longest dwell times aligns with longitudinal findings indicating that positive emotions are more durable than negative ones. For instance, Cohn et al.^30^ found that daily positive affect predicted increased resilience and life satisfaction. Rand et al.^31^ reported that positive affect facilitates cooperation even when negative affect is statistically controlled. Fredrickson’s broaden-and-build theory posits that positive affect initiates “upward spirals” of persistence and resource building^22^. The present HMM-based analysis quantitatively formalizes these claims by estimating positive states as stable attractors in an interactive cultural context. In contrast, neutral states exhibited the lowest self-transition probability and higher entropy, corroborating prior research emphasizing their volatility. Li et al.^32^ observed that external events easily disrupted neutrality on social media, while Stroessner et al.^15^ showed that positive moods reduce perceptions of intergroup differences, suggesting that neutrality is unlikely to endure in socially salient contexts. Collectively, our findings provide probabilistic evidence that neutrality functions as a transitional rather than enduring configuration, consistent with work on affective variability highlighting the susceptibility of neutral or ambivalent states to fluctuation^24^.

Prevalence of positive states. The predominance of positivity in the stationary distribution is consistent with a broad literature on the protective and pervasive role of positive affect. For example, Yin et al.^33^ found that positive tweets outnumbered negative ones during the COVID-19 pandemic, while Cikara et al.^17^ showed that group contexts with weak competitive boundaries foster empathy and collective joy over hostility. Kuranova^16^ similarly conceptualized Resilience as the speed of emotional recovery, with positive dynamics Linked to Reduced psychopathology risk. By quantifying long-run equilibria in the 86.3% range, this study formally estimates emotional attractors in a participatory art environment, providing one of the first empirical demonstrations of positivity as a dominant equilibrium in cultural practice.

Notably, the results highlight not only the durability and dominance of positive affect but also the volatility of neutrality and the transience of negative states. At the same time, they extend the field by integrating probabilistic modelling with participatory art and providing robust quantitative evidence for the persistence of positivity across model specifications and temporal contexts.

### Contributions and novel insights

While this study’s findings align with prior literature emphasizing the durability and prevalence of positive affect, they also yield several novel contributions that extend existing knowledge.

First, the empirical context is distinctive. Previous investigations into affective dynamics have primarily focused on laboratory experiments^22,30^, social media analyses^32,33^, or educational environments. In contrast, this study applies probabilistic modelling to data collected from a participatory public art installation (HappyHere), demonstrating that positivity and resilience theories also manifest within cultural and aesthetic settings. Second, the HMM uncovered a three-state latent structure rather than a binary model. Beyond a low/negative state and a highly positive state, a moderately positive configuration emerged as a stable attractor, whereas the highly positive state functioned as a rarer “ceiling effect.” This tripartite structure affords a more nuanced interpretation than conventional binary valence frameworks. Third, the analysis quantified long-run attractors. Although broad-and-build frameworks describe “upward spirals,” prior work Rarely estimates equilibrium. Here, stationary distributions Revealed that positive states accounted for 86.3% of the long-term equilibrium, offering one of the first attractor-based quantitative estimates in a public art context. Fourth, robustness was established across model specifications and temporal bins. Despite minor differences between morning and afternoon submissions, effect sizes were negligible, underscoring the stability of positive dominance across conditions. Finally, the analysis clarified the role of neutrality. Neutral states exhibited the lowest self-transition probability and higher entropy, functioning as transitional rather than stable configurations. These findings reframe neutrality not as a midpoint on a single continuum but as a passage toward positivity.

These contributions advance affective science by integrating probabilistic modelling with participatory cultural practice, yielding new structural, quantitative, and contextual insights into population-level emotional dynamics.

### Theoretical implications

The results can be interpreted within the interdisciplinary framework introduced in Sect. 3, which integrates computational modelling, psychological theories of affective stability, and interaction design.

First, population-level HMM modelling illustrates the utility of probabilistic frameworks in revealing hidden structures of collective emotion. Even when applied to cross-sectional data, the model recovered differentiation, transition asymmetries, and long-run convergence, highlighting its potential as a structural analogue to temporal processes when actual within-person sequences are unavailable. Despite inherent limitations, adapting HMMs to cross-sectional population data provides a valuable tool for uncovering latent emotional structures that would otherwise remain obscured. Second, the predominance of positive states resonates with established psychological theory. The broaden-and-build framework^13^ posits that positive emotions accumulate cognitive and social resources, fostering resilience. The elevated self-transition probability and extended dwell time of positive states provide probabilistic support for this account. Conversely, the volatility of neutrality is consistent with prior evidence that ambivalent states are unstable and context-dependent^32^. Third, the installation context underscores the role of interaction design as an emotional regulator. Immersive, multisensory participation may reinforce positive affect and reduce the persistence of negativity, echoing earlier research on empathy and prosocial affect in interactive media^19^. Accordingly, the observed convergence toward positivity likely reflects both intrinsic affective dynamics and the moderating influence of designed environments.

These implications demonstrate how computational modelling, affective science, and design research can be productively integrated. They highlight the importance of interpreting emotional stability and volatility as emergent properties shaped jointly by individual tendencies and environmental affordances. These results are also consistent with prior work in art psychology, which shows that participatory and public art reliably elicits positive emotional engagement^34,35^.

### Practical and design implications

Beyond theoretical and methodological contributions, the findings carry actionable implications for designing interactive systems and cultural experiences. Specifically, the observed stability of positive states highlights the potential of participatory art as a medium for emotional well-being. Installations can serve aesthetic purposes and operate as platforms that support resilience and cultivate collective positivity in public settings. Second, the transitional character of neutrality identifies specific design levers. Because neutral states are unstable and tend to drift toward positivity, designers can incorporate subtle cues—such as light-touch multisensory prompts or social affordances—to guide ambivalent participants toward more durable positive engagement. Third, the estimation of long-run attractors provides an implementable evaluation metric. Probabilistic models can assess whether specific design features (e.g., feedback loops, group density, sensory integration) shift the stationary distribution toward greater occupancy of positive states, thereby enabling evidence-guided iteration in cultural practice. Finally, the principles articulated here extend beyond the arts. Fostering positive stability, treating neutrality as transitional, and minimizing sustained negativity are guidelines that can inform the design of educational technologies, digital health systems, and urban public spaces. These applications underscore the broader role of interactive environments as affective regulators that support individual and collective well-being.

### Limitations and future research

Several limitations temper the present findings. First, because the data are cross-sectional. While HMMs can approximate population-level structural tendencies, they cannot substitute for actual within-person temporal sequences. Future studies should employ longitudinal or experience-sampling methods to test the persistence of the identified attractor states. Second, reliance on self-reports narrows the measurement bandwidth. Although the SWEMWBS items provide reliable well-being indicators, they cannot capture physiological or behavioral signatures of affect. Moreover, because these items are framed retrospectively over the previous two weeks rather than capturing momentary states, participants’ reports may incorporate broader life circumstances alongside the immediate installation experience. This retrospective framing further constrains the extent to which the specific influence of HappyHere can be isolated. Integrating multimodal data—such as EEG, heart-rate variability, or movement traces—would deepen the mapping of latent states. Third, the empirical context is limited to a single public installation. Replication across different installations, cultural settings, and design modalities is needed to assess generalizability; comparative Research should test whether positive attractors are universal or contingent upon specific design features. Fourth, model-specification choices may have influenced results. Although robustness checks showed convergence across 3- and 7-feature HMMs, alternative covariance assumptions or a different number of states may reveal further nuances; future work could adopt hierarchical or Bayesian HMMs to capture individual heterogeneity. Finally, causal interpretations remain tentative. The dominance of positive states may reflect psychological tendencies, installation features, or broader contextual factors. Experimental manipulations like altering sensory feedback or group density would help identify and isolate causal mechanisms. Sensitivity analyses confirmed that excluding these anomalous values (< 0.1% of entries) did not materially affect descriptive or inferential results, with differences in means and standard deviations < 0.01.

These limitations suggest the need for longitudinal, multimodal, cross-context, and experimental designs to consolidate the claim that positive affect functions as a stable attractor in participatory environments. Addressing these issues would strengthen construct validity for HMM-based analyses and clarify the boundary conditions under which positivity emerges as a robust equilibrium.

## Conclusion

This study investigates whether and how dynamic probabilistic modelling can reveal stable and transitional emotional states within participatory art environments. Drawing on data from the HappyHere installation, Hidden Markov Models (HMMs) were applied to infer latent affective configurations from in-situ self-reports. The analysis demonstrated that positive affective states emerged as the most stable and persistent, with the highest self-transition probability. This finding supports the broader hypothesis that interactive art can serve as a platform for sustained, population-level emotional well-being. These results address the central research question, demonstrating that dynamic emotional modelling can recover robust affective patterns in cultural participation contexts.

Theoretically, the study contributes to affective science by providing formal estimates of stationary distributions and dwell times, extending the broaden-and-build framework into the domain of interactive art. Methodologically, it demonstrates the feasibility of applying HMMs to population-level self-report data gathered in ecologically valid, non-laboratory settings. Identifying a three-state structure—including a moderately positive attractor and an unstable neutral configuration—offers finer-grained interpretability than binary valence models. It converges with emerging perspectives in emotion-driven design.

The findings yield design implications for affect-aware systems in public, educational, and therapeutic contexts. The stability of positive affect suggests that interactive installations can be strategically designed to support and reinforce sustained emotional trajectories, thereby enhancing individual resilience and collective well-being. This modelling framework also offers a tool for evaluating the effectiveness of such interventions and for guiding the development of adaptive systems that respond to user affect in real time.

Nonetheless, several limitations warrant caution in interpretation. The cross-sectional nature of the dataset constrains causal inference, while reliance on self-report measures limits the granularity of emotional representation. Moreover, the cultural and demographic scope of the participants was relatively narrow, limiting generalizability. Future research should integrate multimodal physiological and behavioural data, employ longitudinal designs, and extend investigations to more diverse and multi-site cultural contexts. Embedding affective modelling within adaptive AI systems could enable personalized emotional feedback, enhancing the therapeutic and educational impact of interactive environments.

In sum, this study underscores the potential of participatory art installations as emotionally intelligent systems. Combining dynamic modelling with user-centred design advances theoretical and applied understandings of how interactive environments can foster sustainable emotional well-being. These insights hold relevance for cultural practice and the broader development of emotion-aware technologies in society.

## Data Availability

The dataset used in this study is publicly available from Mendeley Data: Skelly, Martin; Thomas, Peter (2019), “HappyHere at the National Galleries of Scotland”, Mendeley Data, V1, doi: 10.17632/tpcwm8d7d3.1.

## References

[1] Albers, C. J. & Bringmann, L. F. Inspecting gradual and abrupt changes in emotion dynamics with the Time-Varying change point autoregressive model. *Eur. J. Psychol. Assess.* **36** (3), 492–499. 10.1027/1015-5759/a000589 (2020).

[2] Kragel, P. A., Hariri, A. R. & LaBar, K. S. The Temporal dynamics of spontaneous emotional brain States and their implications for mental health. *J. Cogn. Neurosci.* **34** (5), 715–730. 10.1162/jocn_a_01579 (2021). 10.1162/jocn_a_01787 PMC9026845 34705046

[3] Liu, C. L., Lin, P. H. & Su, K. W. A study of the effects of Spatial arrangements and interactive medias on presence, involvement and emotion in senior visitors within a virtual Art gallery. *Int. J. Sci. Res.* **10** (8), 1223–1231. 10.21275/SR21727151140 (2021).

[4] Kang, H. B. Affective content detection using HMMs. In Proceedings of the eleventh ACM international conference on Multimedia (pp. 259–262). (2003).

[5] Reitsema, A. M., Jeronimus, B. F., van Dijk, M. & de Jonge, P. Emotion dynamics in children and adolescents: A meta-analytic and descriptive review. *Emotion* **22** (2), 374–396. 10.1037/emo0000970 (2022). 34843305 10.1037/emo0000970

[6] Yalçın, İ. & Malkoc, A. The relationship between meaning in life and subjective well-being: forgiveness and hope as mediators. *J. Happiness Stud.* **16** (4), 915–929. 10.1007/s10902-014-9540-5 (2015).

[7] Sudhakar, B. & Bensraj, R. Enhanced Evaluation of Sentiment Analysis for Tamil Text-to-Speech Synthesis using Hidden Semi-Markov Model. *Commun. Appl. Electron. (CAE)*, **3**(6), 13–16. DOI: 10.5120/cae2015651971 (2015).

[8] Wang, K. et al. A multi-country test of brief reappraisal interventions on emotions during the COVID-19 pandemic. *Nat. Hum. Behav.* **5** (8), 1089–1110. 10.1038/s41562-021-01173-x (2021). 34341554 10.1038/s41562-021-01173-x PMC8742248

[9] Kuppens, P., Oravecz, Z. & Tuerlinckx, F. Feelings change: accounting for individual differences in the Temporal dynamics of affect. *J. Personal. Soc. Psychol.* **99** (6), 1042–1060. 10.1037/a0020962 (2010). 10.1037/a0020962 20853980

[10] Houben, M., Van Den Noortgate, W. & Kuppens, P. The relation between short-term emotion dynamics and psychological well‐being: A meta‐analysis. *Psychol. Bull.* **141** (4), 901–930. 10.1037/a0038822 (2015). 25822133 10.1037/a0038822

[11] Klonsky, E. D., Victor, S. E., Hibbert, A. S. & Hajcak, G. The multidimensional emotion questionnaire (MEQ): rationale and initial psychometric properties. *J. Psychopathol. Behav. Assess.* **41** (3), 409–424. 10.1007/s10862-019-09741-2 (2019).

[12] Rabiner, L. R. A tutorial on hidden Markov models and selected applications in speech recognition. *Proc. IEEE*. **77** (2), 257–286. 10.1109/5.18626 (1989).

[13] Fredrickson, B. L. What good are positive emotions? *Rev. Gen. Psychol.* **2** (3), 300–319. 10.1037/1089-2680.2.3.300 (1998). 21850154 10.1037/1089-2680.2.3.300 PMC3156001

[14] Ashby, F. G., Isen, A. M. & Turken, A. U. A neuropsychological theory of positive affect and its influence on cognition. *Psychol. Rev.* **106** (3), 529–550. 10.1037/0033-295x.106.3.529 (1999). 10467897 10.1037/0033-295x.106.3.529

[15] Steven, J., Stroessner, D. M., Mackie & Venezia Michalsen. Positive Mood and the Perception of Variability Within and Between Groups. Group Processes and Intergroup Relations, 8 (1), pp.5–25. ff10.1177/1368430205048619 ff. (2005). ffhal-00571594

[16] Kuranova, A. There and back again: a dynamical perspective on psychological resilience. [Thesis fully internal (DIV), University of Groningen]. University of Groningen. (2022). 10.33612/diss.223532259

[17] Cikara, M. et al. November. Their Pain Gives Us Pleasure: How Intergroup Dynamics Shape Empathic Failures and Counter-Empathic Responses. Journal of Experimental Social Psychology 55 : 110–125 © 2014 Elsevier Inc. (2014). 10.1016/J.JESP.2014.06.007 10.1016/j.jesp.2014.06.007 PMC4112600 25082998

[18] Dreisbach, G. & Goschke, T. Positive affect modulates cognitive control: reduced perseveration at the cost of increased distractibility. *J. Experimental Psychology: Learn. Memory Cognition*. **30** (2), 343. 10.1037/0278-7393.30.2.343 (2004). 10.1037/0278-7393.30.2.343 14979809

[19] Isbister, K. *How Games Move Us: Emotion by Design* (MIT Press, 2016).

[20] Seong, S. et al. Impact of virtual reality–based biofeedback on sleep quality among individuals with depressive symptoms, anxiety symptoms, or both: A 4-week randomized controlled study. *J. Med. Internet. Res.* **27** (1). 10.2196/65772 (2025). Article e65772. 10.2196/65772 PMC12204043 40539943

[21] Tang, G. Y. & Goh, Y. W. Modelling the impact of sensory marketing on impulse buying: an empirical study using PLS-SEM. *J. Retailing Consumer Serv.* **71**, 103192. 10.1016/j.jretconser.2022.103192 (2023).

[22] Fredrickson, B. L. The role of positive emotions in positive psychology: the broaden-and-build theory of positive emotions. *Am. Psychol.* **56** (3), 218. 10.1037/0003-066x.56.3.218 (2001). 11315248 10.1037//0003-066x.56.3.218 PMC3122271

[23] Shodeinde, A. O., Ogunnaike, O. O. & Kehinde, O. J. Assessing the impact of organizational capabilities on sustainable performance in the Nigerian manufacturing sector. *J. Manuf. Technol. Manage.* **34** (3), 456–477. 10.1108/JMTM-05-2022-0187 (2023).

[24] Kuppens, P., Oravecz, Z. & Tuerlinckx, F. Feelings change: accounting for individual differences in the Temporal dynamics of affect. *J. Personal. Soc. Psychol.* **99** (6), 1042–1060. 10.1037/a0020962 (2010). 10.1037/a0020962 20853980

[25] Oli, D. A., Khadka, A. & Maharjan, S. Impact of environmental anxiety on conflict mediation and pro-environmental behavior: A moderated mediation analysis. *J. Environ. Psychol.* **93**, 102234. 10.1016/j.jenvp.2024.102234 (2024).

[26] Leal, C. T. F., de Araújo, C. C. S., Júnior, S. B. B. & de Sousa, F. J. M. Influence of external capabilities and dynamic capabilities on innovation behavior in Brazilian industrial companies. *Innov. Manage. Rev.* **20** (1), 70–86. 10.1108/INMR-07-2021-0123 (2023).

[27] Diener, E., Lucas, R. E. & Oishi, S. Advances and open questions in the science of subjective well-being. *Collabra: Psychol.* **4** (1), 15. 10.1525/collabra.115 (2018). 30637366 10.1525/collabra.115 PMC6329388

[28] Skelly, M. & Thomas, P. Reflections on the design of HappyHere, a digital installation facilitating participation, reflection, and discussion on well-being data in the Arts sector. In Design4Health Conference 2024. (2024).

[29] Skelly, M. & Thomas, P. HappyHere at the National galleries of scotland, Mendeley data, V1. (2019). 10.17632/tpcwm8d7d3.1

[30] Cohn, M. A., Fredrickson, B. L., Brown, S. L., Mikels, J. A. & Conway, A. M. Happiness unpacked: positive emotions increase life satisfaction by Building resilience. *Emotion* **9** (3), 361. 10.1037/a0015952 (2009). 19485613 10.1037/a0015952 PMC3126102

[31] Rand, D. G., Kraft-Todd, G. & Gruber, J. The collective benefits of feeling good and letting go: positive emotion and (dis)inhibition interact to predict cooperative behavior. *PLoS ONE*. **10** (1), e0117426. 10.1371/journal.pone.0117426 (2015). 25625722 10.1371/journal.pone.0117426 PMC4308081

[32] Li, T., Wang, X., Yu, Y., Yu, G. & Tong, X. Exploring the dynamic characteristics of public risk perception and emotional expression during the COVID-19 pandemic on Sina Weibo. *Systems* **11** (1), 45. 10.3390/systems11010045 (2023).

[33] Yin, H., Yang, S. & Li, J. Detecting topic and sentiment dynamics due to COVID-19 pandemic using social media. In *International conference on advanced data mining and applications* (pp. 610–623). Cham: Springer International Publishing. (2020)., November 10.1007/978-3-030-65390-3_46

[34] Kelleher-Clarke, A., Bartoli Jones, A. & Omigie, D. Exploring barriers to and drivers of participatory arts engagement in early adolescence. *Psychol. Aesthet. Creativity Arts*. 10.1080/17533015.2022.2035417 (2023).

[35] Pelowski, M., Specker, E., Gerger, G., Leder, H. & Weingarden, L. S. Do you feel like I do? A study of spontaneous and deliberate emotion sharing and Understanding between Artists and perceivers of installation Art. *Psychol. Aesthet. Creativity Arts*. **14** (3), 276. 10.1037/aca0000201 (2020).

